# Application of an organotypic ocular perfusion model to assess intravitreal drug distribution in human and animal eyes

**DOI:** 10.1098/rsif.2021.0734

**Published:** 2022-01-26

**Authors:** D. Chan, G. J. Won, A. T. Read, C. R. Ethier, E. Thackaberry, S. R. Crowell, H. Booler, V. Bantseev, J. M. Sivak

**Affiliations:** ^1^ Donald K Johnson Eye Institute, Krembil Research Institute, University Health Network, Toronto, Ontario, Canada; ^2^ Wallace H. Coulter Department of Biomedical Engineering, Georgia Institute of Technology and Emory University School of Medicine, Atlanta, Georgia, USA; ^3^ Safety Assessment, Genentech Inc., San Francisco, CA, USA; ^4^ Preclinical and Translational Pharmacokinetics and Pharmacodynamics (PTPK) Genentech Inc., San Francisco, CA, USA; ^5^ Department of Ophthalmology and Vision Science, University of Toronto, Toronto, Ontario, Canada; ^6^ Department of Laboratory Medicine and Pathobiology, University of Toronto, Toronto, Ontario, Canada

**Keywords:** ocular perfusion, outflow facility, drug delivery, pharmacokinetics, intravitreal drug development

## Abstract

Intravitreal (ITV) drug delivery is a new cornerstone for retinal therapeutics. Yet, predicting the disposition of formulations in the human eye remains a major translational hurdle. A prominent, but poorly understood, issue in pre-clinical ITV toxicity studies is unintended particle movements to the anterior chamber (AC). These particles can accumulate in the AC to dangerously raise intraocular pressure. Yet, anatomical differences, and the inability to obtain equivalent human data, make investigating this issue extremely challenging. We have developed an organotypic perfusion strategy to re-establish intraocular fluid flow, while maintaining homeostatic pressure and pH. Here, we used this approach with suitably sized microbeads to profile anterior and posterior ITV particle movements in live versus perfused porcine eyes, and in human donor eyes. Small-molecule suspensions were then tested with the system after exhibiting differing behaviours *in vivo*. Aggregate particle size is supported as an important determinant of particle movements in the human eye, and we note these data are consistent with a poroelastic model of bidirectional vitreous transport. Together, this approach uses ocular fluid dynamics to permit, to our knowledge, the first direct comparisons between particle behaviours from human ITV injections and animal models, with potential to speed pre-clinical development of retinal therapeutics.

## 1. Introduction

The recent development of intravitreal (ITV) therapeutics for retinal diseases has established intraocular drug delivery as a new cornerstone for retinal care [1–4]. Thus, intraocular delivery systems and formulations hold immense promise as powerful platforms for retinal therapeutics [5–7]. However, as new treatments are developed, a major translational challenge has arisen; predicting and optimizing the disposition of ITV drugs and their excipients for the human eye [8,9]. One prominent, but poorly understood issue often observed in pre-clinical toxicology studies has been the anterior movement of drug or excipient particles from the vitreal compartment to the anterior chamber (AC) [10,11]. These outcomes are consistent with clinical observations of migrating pigment or retinal debris in the same direction [12–14]. The accumulation of particles in the AC carries a serious risk of physical toxicity, and the potential to occlude the outflow of aqueous fluid through the trabecular meshwork located at the iridocorneal angle. The reduced outflow of aqueous fluid can rapidly increase intraocular pressure (IOP), a major risk factor for the development of glaucoma [15–17]. Yet, the factors influencing this issue have been surprisingly difficult to study owing to critical challenges. Key anatomical differences in intraocular barriers, vitreous composition, and fluid flow between human and common animal model eyes make for uncertain clinical predictions, and the inability to obtain ocular samples from clinical subjects means it has not been possible to determine the human equivalent data. Therefore, clinical scaling for ITV particle distribution remains largely unknown, and there is a lack of established *in vitro* or *ex vivo* models to predict and systematically investigate these questions.

The parameters governing particle movements following ITV administration are not well understood or predicted with confidence. Yet, the dynamics of aqueous fluid at the front of the eye has been extensively described [18–22]. Aqueous humour is produced by the ciliary processes into the posterior chamber and flows anteriorly through the pupil to exit at the iridocorneal angle through the sieve-like trabecular meshwork. In the human eye, this primary route accounts for the majority of aqueous flow, along with secondary uveoscleral routes [23], and generates a current of 2–3 µl min^−1^ to maintain IOP in combination with outflow resistance [23–25]. However, these parameters are species specific [26]. In comparison, the dynamics of the vitreous body and its interactions with anterior compartments and retina are not as well studied. The vitreous body is a complex avascular gel primarily composed of a network of collagen and hyaluronic acid. Although the main vitreous components are conserved, the relative ratios differ greatly between species and with age, producing dramatically different viscoelastic properties and gel/liquid ratio (rheology) [27,28], and posing a challenge to predictive and experimental modelling. For example, the rabbit eye exhibits a highly viscous vitreous and altered aqueous outflow compared to human and non-human primate (NHP) eyes [28–30]. Nevertheless, studies and modelling of vitreous outflow in multiple species have demonstrated an anterior vitreous convection that is related to the aqueous humour outflow [24,31,32] and is influenced by a declining temperature gradient between posterior and anterior segments [33]. Computational modelling has also predicted retrograde vitreous currents, driving flow towards the central retina [34,–36]. Yet, the disposition of ITV formulations in the human eye in the context of vitreous convection remains unclear, and the allometric relationships between species have not been determined.

Genentech recently encountered anterior particle movements during pre-clinical development of the small molecule GNE-947 as a potential treatment for macular degeneration. GNE-947 was formulated in methylcellulose (MC) as a suspension, with its low-aqueous solubility used to enable sustained exposure to the back of the eye, similar to the commercially available long-acting steroid formulation Triesence (Alcon). However, following ITV injection in cynomolgus monkeys, GNE-947 particles were observed to rapidly accumulate in the AC, settling in the angle, and ultimately raising IOP. However, no comparable movement of Triesence was detected in rabbit eyes, nor has been reported clinically. This inconsistency in particle movements raised complex toxicological and pharmacokinetic challenges that required a new model capable of reproducing these observations in the context of intraocular compartments and barriers. Organotypic *ex vivo* ocular perfusion techniques have been previously developed by us and others to assess aqueous outflow in relation to IOP and glaucoma [37–42]. These approaches re-establish anterior flow with a synthetic aqueous substitute to mimic fluid dynamics in the anterior segment and preserve outflow tissue viability and pharmacology in various vertebrate and even human eyes [37,42–46]. Yet, to our knowledge, the influence of this homeostatic flow and pressure has not been studied in relation to the vitreous and retina, or used to study ITV injections or particle movements. In comparison, posterior artery cannulations have been developed to study drug distribution in the retina [47–50], but these do not produce defined intraocular flow to address intercompartmental particle movements, nor are these methods possible in human eyes owing to inconsistent and irreversible coagulation of retinal capillaries. Therefore, many of these relationships for ITV drug distribution remain surprisingly unknown.

Here, we present a series of optimizations and validations, first in porcine and then human and NHP eyes, of a whole globe anterior ocular perfusion approach to study ITV particle distribution and movements. First, the model was optimized for purposes of ITV injection assessments, then anterior particle movements were assessed using labelled microbeads of relevant sizes, finally the system was validated against the *in vivo* results with GNE-947 and Triescence. To our knowledge, these data perform the first direct comparisons between particle behaviours from animal model and human ITV injections, providing a foundation for refined modelling comparisons, and a new platform for drug investigations.

## 2. Material and methods

### 2.1. Tissue preparation

**Figure 1.**
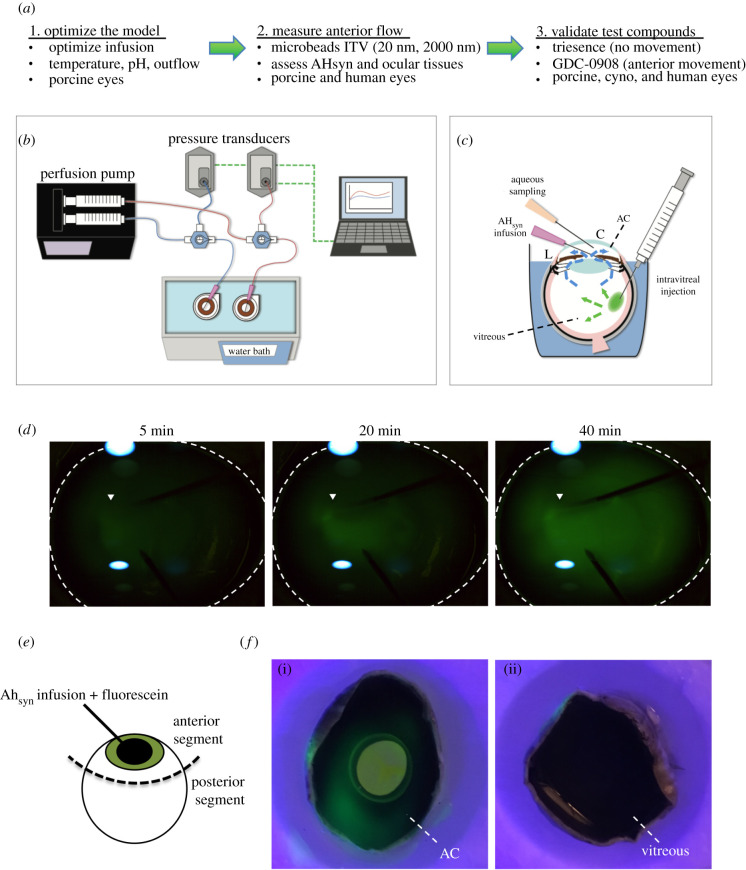
System design and establishment of homogeneous anterograde convection. (*a*) An overview outlining the experimental approach. (*b*) A cartoon schematic of the system shows a syringe pump infusing two eyes cannulated into their posterior chambers and maintained at a constant temperature in a water bath. The infusion lines incorporate three-way valves attached to transducers to monitor pressure at the point of cannulation. The transducers are connected to a data logger for storage and readout. (*c*) A cartoon cross-section of an eye mounted in media to the limbus, leaving the cornea exposed. The posterior chamber cannulation for AH_syn_ infusion behind the iris, the aqueous sampling port in the AC and the ITV injection are all indicated. Dashed-line arrows indicate fluid flow (blue) and particle dispersion (green). See the electronic supplementary material, figure S6 for a more detailed diagram of the eye and its compartments (AC; anterior chamber, C; cornea, L; corneal limbus). (*d*) Whole eye photographs showing fluorescein introduced into the infusion line cannulating the posterior chamber over time (arrowhead), resulting in a steady increase in fluorescence that filled the AC by 40 min (dashed line, *n* = 3). (*e*) Following perfusion, eyes were carefully bisected posterior to the limbus (dashed line) to separate the anterior and posterior segments, the lens and iris removed to expose the AC, and illuminated with a fluorescent lamp. (*f*) Representative images of fluorescein localized to the anterior segment (i), with no fluorescence visible in the posterior segment (ii), demonstrating the anterograde nature of flow and the maintenance of barriers between ocular compartments (*n* = 3, AC; anterior chamber).

Fresh porcine eyes were obtained from the University Health Network animal resource centre within 6 h (h) of enucleation. For live pig studies, adult Yorkshire pigs were anaesthetized with 2.5% isofluorane for ITV injections as described below. All animal experiments were performed in accordance with the ARVO Statement for the Use of Animals in Ophthalmic and Vision Research and according to approved UHN Animal Use Protocols. Donated human eyes were obtained through the Eye Bank of Canada (Ontario Division) and were intact healthy globes from donors aged from 22 to 67 years. Eyes were enucleated within 12 h and the tissue was received within 24 h of the time of death. Eyes with history of ocular tumours, vascular disease or glaucoma were excluded. Human eyes were prepared, perfused and assessed similarly to porcine eyes. All experiments using human tissue were performed according to protocols approved by the UHN Research Ethics Board and adhered to the Declaration of Helsinki. For some experiments, cynomolgus monkey (cyno) eyes were donated by Charles River Labs and received on ice within 24 h of enucleation. For all eye preparations: immediately upon receiving the enucleated eyes, extraocular tissues were carefully removed and the eyes were wrapped in compression gauze around the global equator. Wrapped eyes were oriented, anterior-side up, in 50 ml beakers and then immersed to the level of the limbus in a bathing buffer of sterile Dulbecco's phosphate buffered saline (DPBS) (Sigma-Aldrich) supplemented with 5.5 mM glucose. Beakers containing the eyes were then placed into a heated water bath prior to cannulation. Eyes were cannulated by puncturing a 25G needle (BD Vacutainer®) through the cornea, 2 mm anterior to the limbus. The needle was then guided to the posterior chamber by threading carefully through the gap between the iris and the lens. Eyes were also cannulated at the AC for collection of aqueous samples. (Note: additional details for the placement of cannulations and ocular anatomy details are presented in figure 1 and the electronic supplementary material, figure S6).

### 2.2. Ocular perfusion system

A two-channel system was built that included a motorized pulse-free syringe pump (PHD 2000, Harvard Apparatus) and dual pressure sensors, so that two eyes could be assessed simultaneously. The pump drove two 10 ml syringes loaded with infusion buffer of degassed DPBS supplemented with 5 mM glucose as a simple synthetic aqueous humour equivalent (AH_syn_) into each eye at constant rate of 2.4 µl min^−1^ for the indicated time (note this is identical to the bathing buffer). The perfusate was passed through a 0.2 µm syringe filter installed between each syringe and an infusion line of PE160 polyethylene tubing (Intramedic®, Becton Dickinson). Each infusion line was split by a three-way valve (Hamilton®) to incorporate a calibrated pressure sensor/transducer (142PC01G, Honeywell) to monitor pressure at the point of cannulation (electronic supplementary material, figure S1). Transducer output signals in voltage were recorded with a portable multi-channel data logger (OM-DAQPRO-5300, Omega). Outflow facility (C) was calculated according to a simplified formula for enucleated eyes, C = Flow/IOP, based on [43], and as per our previous publications with whole globe perfusion for anterior outflow measurements [37,38,51,52]. For the present purposes, the flow rate was kept at a constant 2.4 µl min^−1^ rather than constant pressure.

### 2.3. Optimization of physiological parameters

For temperature assessment studies, thermocouple subminiature hypodermic probes (HYP1, Omega), with a 30 gauge (×15 mm) needle size, were chosen for their small size, fast response rate and accuracy to ±0.1°C at 37°C. Probes were carefully inserted into the AC through the cornea and vitreous chamber through the sclera (3 mm posterior to the limbus). These probes were connected to the same portable data logger for storage and readout. The internal ocular temperatures of the anterior and vitreous chambers were measured with increasing water bath temperatures in equal increments from 37°C to 38.5°C in order to establish the optimal temperature settings. To measure intraocular pH, microelectrodes (9863BN, Orion), with 16 gauge stainless steel needle tips and precision of 0.02, were inserted into the eyes in a similar fashion to the temperature probes. To validate outflow tissue viability, whole eyes undergoing active AH_syn_ perfusion were injected with 300 µl of 10 µM Cytochalasin D (StressMarq Biosciences Inc.) into the AC after 2 h of stable perfusion, and the resulting tissue response was assessed by monitoring the change in outflow facility (calculated from pressure readings as described above), as compared to the contralateral eye undergoing stable perfusion. To assess fluorescein distribution, whole-globe perfusions were carried out with 0.25% (w/v) fluorescein dye (Sigma-Aldrich) in the perfusate. ACs were visualized using a Leica M80 stereo fluorescent microscope. Images were taken at 5, 20 and 40 min of perfusion. After 40 min, eyes were removed from the system, hemi-dissected through the equator, and anterior and posterior segments were imaged, vitreal-side up.

### 2.4. Intravitreal injections of microbeads

A 50 µl 1 : 1 suspension of 2% stock mixtures of 20 nm (4.143 × 10^13^ beads ml^−1^, green) and 2000 nm (1.326 × 10^8^ beads ml^−1^, red) fluorescent polystyrene microbeads (F8787 and F8826, respectively; FluoSpheres™, ThermoFisher) was prepared and loaded into a 1 ml luer-locking syringe with a 30G½ needle. Once a steady baseline perfusion was achieved, a volume equivalent to the injection was first slowly removed using a cannulation reservoir. Then, a syringe loaded with the microbead formulation was inserted through the inferior sclera, 3 mm posterior to the limbus. The microbead mixture was then slowly injected into the lateral inferior vitreous over 1 min. To prevent backflow of injectate, the needle was held in place for 1 min after injection before gentle withdrawal from the eye. Following ITV injection, the perfusion system ran for 4 h, with passive aqueous sampling at 2 h and 4 h time-points of 30–40 µl through a second cannulation without disrupting the infusion flow, after which a reservoir was slightly raised to rapidly repressurize and refill the AC. For live pig studies, animals were anesthetized and placed in either a supine or pronate position, and then intravitreally injected with 50 µl of mixed microbeads in a similar fashion to the excised pig eyes. Pigs were kept under sedation for 4 h after injection, allowing the fluorescent microbeads to distribute *in vivo*, followed by euthanasia and enucleation. In this case, a final 50 µl sample of aqueous humour was withdrawn at 4 h for analysis of microbead concentrations.

### 2.5. Analyses of microbead distribution

Following perfusions, eyes were slit near the limbus and immersion fixed overnight in 4% (v/v) paraformaldehyde, then hemi-dissected through the equator, cryoprotected with 30% (w/v) sucrose solution and embedded in optimal cutting temperature medium (OCT) (Sakura) for cryosectioning. Both anterior and posterior segments were sectioned at 18 µm thickness, taking care to maintain a lower than usual temperature (−25°C) to ensure firm embedding media and cut consistently in a direction to minimize potential bead displacement. Sections were compared before and after mounting to ensure a consistent bead signal. Retinal sections were not washed, but were stained with 4',6-diamidino-2-phenylindole (DAPI) (ThermoFisher) during mounting for visualization of nuclear layer organization, while anterior segment drainage tissues were identified with an antibody against CD31 (Abcam) to identify the angular aqueous plexus (pig) and Schlemm's canal (SC) (human) endothelial cells. Retinal cell types were assessed with primary antibodies against RNA-binding protein with multiple splicing (RBPMS; Phosphosolutions), CHX10 (Exalpha), R/G-opsin (Abcam) and glutamine synthetase (GS; Abcam). Sections and projections were imaged on a Nikon Ti2 Confocal microscope using identical settings between images for the green and red channels as previously reported [53,54]. For aqueous fluid samples: the number of microbeads per ml of the stock green (0.02 µm) and red (2 µm) fluorescent microbeads was determined using the formula: microbeads perml = 6C × 10¹²/*ρ*π*φ*³ (where C = the concentration of suspended beads in g ml^−1^, *ρ* = the density of the microbead polymer in g ml^−1^ and *φ* = the bead diameter in micrometers). From a stock 1 : 1 (green : red) microbead mixture, a series of serial dilutions were made in order to establish a standard fluorescence curve. Subsequently, 30 µl aqueous samples from perfused, live or non-perfused eyes were loaded onto a 384-well microplate and compared to the standard curve. Fluorescence intensity readings were taken using a CLARIOstar microplate reader (BMG LABTECH). Bead counts were compared using two-way analysis of variance and Bonferonni post hoc multiple comparisons test using Graphpad Prism 9 software.

### 2.6. *In vivo* GNE-947 and triesence study

A proprietary ITV sustained-release formulation of GNE-947 was supplied by Genentech. GNE-947 is a novel and potent small-molecule inhibitor of phosphoinositide 3 kinase/mammalian target of rapamycin (PI3 K/mTOR) formulated as a milled suspension of 10–20 µm particles in 0.5% MC, 0.015% polysorbate 80, 10 mM phosphate buffer and 0.838% sodium in water. The low-aqueous solubility of GNE-947 (approx. 1 µM) combined with the MC-based formulation at 6 or 20 mg ml^−1^ results in a suspension designed for sustained drug release in vitreous humour for periods of 4–6 months. Triesence (Alcon, commercially available) is an injectable flocculent suspension of 40 mg ml^−1^ triamcinolone acetonide in 0.5% carboxymethylcellulose sodium and 0.015% polysorbate 80 in a balanced salt solution, with a reported particle size of 5–10 µm in diameter [55,56], and was used as a comparator. Naïve male cynomolgus monkeys of Chinese origin (Covance Research Products, Inc.) were used in single-dose ITV administration studies. Animals were housed in the animal facilities under standard conditions. The local animal care committee (Covance Laboratories Inc.), in accordance with the Association for Research in Vision and Ophthalmology's Statement for the Use of Animals in Ophthalmology and Vision Research, approved all animal protocols. Animals were approximately 3 to 5 years of age and ranged in weight from 2.2 to 4.2 kg. For *in vivo* studies, 50 µl of each formulated test article was injected intravitreally into both eyes (three animals for GNE-947 and six animals for Triesence). Briefly, ITV injections were performed by a board-certified veterinary ophthalmologist. Prior to the ITV injections, animals were anesthetized with intramuscular injections of ketamine/dexmedetomidine. After aseptic preparation of the ocular surface, each eye received 50 µl ITV injections. A topical antibiotic (Tobrex®) was instilled in each eye following dosing on day 1 and animals were monitored for up to four weeks. Assessment of ocular tolerability was based on ophthalmology examinations (OE; slit lamp biomicroscopy and indirect ophthalmoscopy), IOP and fundus photography. Particulate matter observed in the AC and iridocorneal angle was assessed by gonioscopy.

### 2.7. *Ex vivo* drug particle migration analyses

GNE-947 and Triesence formulations were injected in a similar location as the live cyno study into enucleated human, cyno and porcine eyes, followed by 4 h of perfusion as described for microbeads above. The aggregate particle size of formulated GNE-947 and Triesence suspensions used for injection was measured by spotting on coverslips, followed by the analysis of equivalent diameter using a Nikon Ti2 microscope and NIS analyses software (electronic supplementary material, figure S4). Following perfusion, 60 µl aqueous was collected and concentrated 10 × by spinning at 12 000 rpm for 10 min at 4°C, and resuspending to 6 µl. Samples were then mounted on slides with coverslips and imaged on a Nikon confocal microscope with 488 nm excitation, which we had determined to induce particle autofluorescence (electronic supplementary material, figure S5). Drug particles were counted from at least three images per sample and averaged to generate final numbers. Differences between drug particle numbers for each species was analysed by two tailed *t*-test with Graphpad Prism 9 software.

## 3. Results

### 3.1. System design and physiological optimizations

The overall study design and approach are outlined in figure 1*a*. Our whole-globe ocular drug perfusion approach involves a dual-channel set-up with a syringe pump infusing AH_syn_ media (5 mM glucose supplemented DPBS) into enucleated eyes through cannulations, based on our previous publications with whole-globe perfusion for anterior outflow measurements [37,38,51,52]. The in-line connection of pressure transducers with three-way valves enables continuous pressure monitoring through a laptop computer (figure 1*b*). The eyes were immersed in a beaker containing the same glucose-DPBS buffer raised to the level of the limbus and maintained at a constant temperature in a water bath, leaving the cornea exposed to atmospheric temperatures (figure 1*c*). A Kimwipe was placed on top of the exposed cornea to absorb surrounding buffer in order to prevent dehydration. The AH_syn_ was infused into the posterior chamber (located in the anterior segment), and the AC received an aqueous sampling port, leaving the vitreous chamber available for drug administration (figure 1*c*). (Note: detailed anatomy of the eye is presented in the electronic supplementary material, figure S6).

Our initial experiments were directed towards adaptation and optimization of the system to reproduce *in vivo* physiological conditions as closely as possible. These were performed in fresh porcine eyes, which serve as a close human anatomical surrogate in many respects [57–60]. The system's ability to monitor ocular integrity was assessed by continuous monitoring of aqueous outflow facility, calculated from calibrated pressure transducer readings (electronic supplementary material, figure S1). A constant flow rate of 2.4 µl min^−1^ was established from the syringe pump reservoir, corresponding to average aqueous flow measurements [23–25]. In healthy porcine globes, this infusion resulted in a stable physiologic pressure and calculated outflow facility (electronic supplementary material, figure S2). A viable anterior tissue response was confirmed by injecting cytochalasin D into the AC, which has been previously reported to increase outflow through the trabecular meshwork [61,62] (electronic supplementary material, figure S2). To determine eyes acceptable for study, we set an inclusion range for outflow facilities, the ratio of flow to driving pressure difference, that were stably maintained between 0.35 and 0.18 µl min^−1^/mmHg^−1^, which corresponds to physiological IOPs, absent the contributions of episcleral venous pressure (EVP) *in vivo* [43,45]. Approximately 15% of eyes failed these criteria for a combination of technical and enucleation-related reasons, and so were not used in our studies. Once physiological pressures had been achieved for 30 min, the temperature of the water bath and the pH of the AH_syn_ were measured and optimized by monitoring these parameters in the aqueous and vitreous (electronic supplementary material, figure S3). Importantly, the exposed cornea accurately reproduces a critical anterior : posterior temperature differential that contributes to the anterograde convection current against an average room temperature of 20°C [33,63] (electronic supplementary material, figure S3A). A water bath setting of 38°C was chosen to accurately reproduce these *in vivo* differential temperatures [33]. After 4 h at this setting, physiological pH was maintained in the aqueous and vitreous chambers when compared to freshly enucleated eyes, indicating a lack of acidosis and suggesting the maintenance of general cellular metabolism in the globe (electronic supplementary material, figure S3B). Therefore, these settings were used for subsequent studies once a 30 min stable perfusion had been established.

To confirm consistent anterior flow in the ocular perfusion system, fluorescein was added to the AH_syn_ perfusate, and the AC was imaged in time-lapse by fluorescent stereomicroscope. Fluorescent images demonstrated that in the first 5 min of perfusion, fluorescein begins to distribute into the AC from the cannulation site in the posterior chamber and increases in extent with time. After 40 min of perfusion, fluorescein was detected homogeneously throughout the AC (figure 1*d*). Following the fluorescein perfusion, eyes were hemi-dissected at the equator (figure 1*e*), and both anterior and posterior segments were imaged under fluorescent illumination. Stereomicroscope images showed fluorescent signal exclusively in the anterior segment, with no signal present in the vitreous compartment (figure 1*f*). These data indicate an anterograde fluorescein flow from the posterior chamber cannulation and suggest that intraocular compartments remain intact.

### 3.2. Intraocular particle distribution is similar between live and perfused eyes

**Figure 2.**
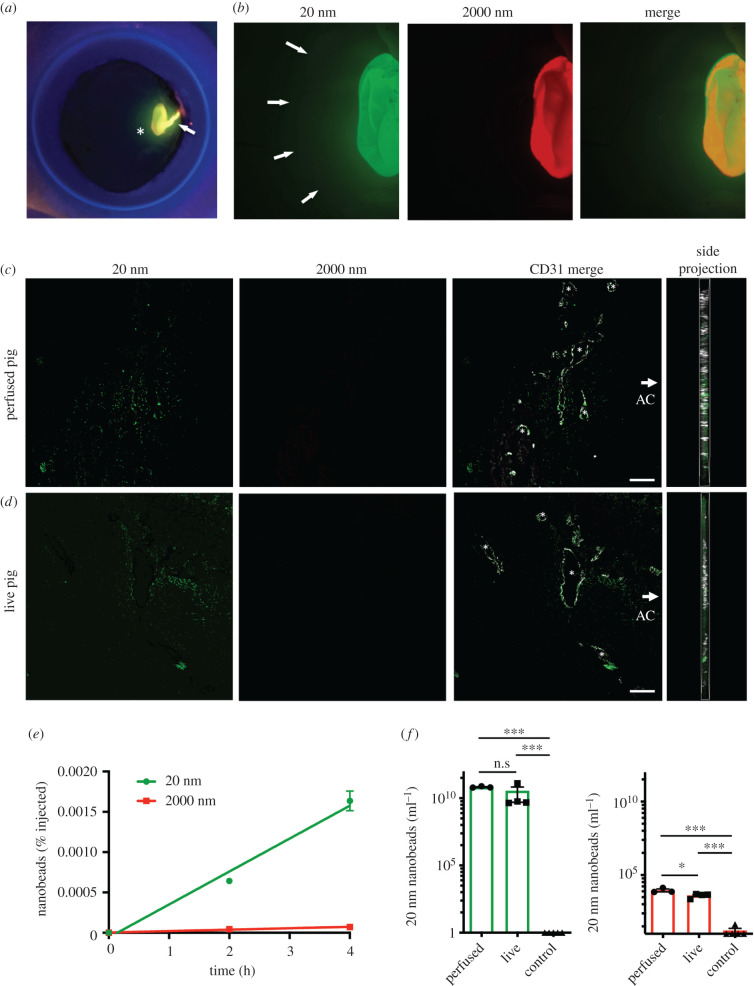
Anterior bead distribution as a function of size is conserved between live and perfused pig eyes. (*a*) Representative image of a yellow pellet (asterisk) and needle trace (arrow) after 4 h of perfusion indicates where two sizes of fluorescent microbeads, 20 nm (green) and 2000 nm (red), were deposited into the inferior vitreous in the dissected posterior eye cup (*n* = 3). (*b*) Representative higher magnification stereomicroscope images suggested the 20 nm beads (green) dispersed more than the 2000 nm size (red), visible as a green corona around the pellet (arrows). (*c*) Outflow tissues in sections through the anterior segment were assessed in confocal projections following ITV microbead injections. Only the smaller 20 nm beads (green) were visible in the trabecular meshwork and angular aqueous plexus (asterisks), which was co-stained for CD31 (white, merge), while the larger 2000 nm beads (red) did not appear in this compartment (*n* = 3, arrow indicates the location of the AC for orientation). Note that the orientation and location of the probed tissues are also indicated in the electronic supplementary material, figure S6. A corresponding z-stack side projection was also assessed to ensure beads remained within the sectioned tissue plane. (*d*) Anterior microbead distribution patterns were also observed 4 h following injections in live pig eyes showing similar 20 nm bead depositions in outflow tissues (green), but no 2000 nm beads (red) (n = 3). (*e*) Corresponding fluorescent bead numbers were measured from serial aqueous fluid samples from the same perfused eyes collected at 2 h and 4 h following ITV bead injection and normalized as a percentage of injected beads. A progressive time-dependent increase in 20 nm microbeads was observed, but 2000 nm microbeads remained near baseline (*n* = 3, linear regression shown, note; error bars smaller than the data point do not plot). (*f*) Raw microbead numbers measured from aqueous fluid samples at 4 h from the live pig study were compared with 4 h aqueous fluid samples from perfused *ex vivo* pig eyes, indicating comparable ratios between the 20 nm and 2000 nm sizes (n = 4). There were dramatically higher concentrations of 20 nm beads than 2000 nm in both cases. However, there was a small but significant difference between live and perfused 2000 nm samples. In comparison, controls with no active perfusion (control) showed virtually no aqueous signal detected (*n* = 4). (Scale bars indicate 50 µm, data are mean ± s.e.m., ***p < 0.005, *p < 0.05, n.s.; not significant).

We recently reported on analyses suggesting particle size is an important variable in determining vitreous distribution of biologic agents in the rabbit eye [64]. Therefore, we hypothesized that a similar concept may influence the distribution of other ITV molecules. As a pilot study to visualize anterior distribution of intraocular particles, we used ‘FluoSpheres’, fluorescent polystyrene microbeads of near-neutral buoyancy in physiological fluids (s.g. = 1.055), to perform proof-of-principle experiments that would compare the disposition of two sizes. For this purpose, we selected 20 nm (green) and 2000 nm (red) beads, corresponding to a range on either side of a general 500 nm limit proposed for vitreous diffusion [65,66], and also corresponding to the range of predicted and observed aggregate particle sizes of formulated GNE947 and Triesence suspensions (electronic supplementary material, figure S4). Each microbead was tagged with distinct fluorophores that could be mixed and easily differentiated microscopically. Beads were combined and injected into the inferior central vitreous of porcine eyes, mimicking a common ITV injection location for drug formulations, followed by 4 h of perfusion. Following perfusion, eyes were hemisected and the posterior segment and intact vitreous were analysed under low magnification fluorescent stereomicroscopy. These analyses demonstrated the merged green and red bead signal as a yellow deposit and needle trace (figure 2*a*). With slightly higher magnification the smaller 20 nm beads displayed a green corona, while the larger 2000 nm (red) beads did not, suggesting increased dispersion of the smaller particles (figure 2*b*).

Following careful sectioning, confocal microscopy was used to render higher magnification three-dimensional projections to highlight the anterior distribution of microbeads to the outflow tissues. To minimize the chance of microbead movements during processing, relatively thick (18 µm) cryosections were made at lower than standard temperature, and mounted on gelatin precoated slides. No washing was performed, and the sections were checked under a microscope to ensure undisturbed signals before and after mounting in a medium containing DAPI. Only the small 20 nm (green) microbeads were visible in the trabecular meshwork and pig angular aqueous plexus (analogous to SC), suggesting this size dispersed in an anterior direction, but not the larger 2000 nm beads (red) (figure 2*c*). For each section, a side projection was also analysed to confirm the microbeads remained within the tissue plane. To provide critical validation that these results represent physiologically relevant distribution patterns, we set up a parallel experiment in live pig eyes. Fortunately, we were able to perform this experiment on the same cohort of animals and location as our perfusion samples, except in this case the intraocular injections were performed on anaesthetized pigs prior to scheduled euthanasia. To mimic the perfusion study, 4 h following ITV injections the eyes were enucleated and immersion fixed as previously. Retinas were then sectioned and analysed by confocal projection. Similar to the perfused eyes, the live pig injections showed anterior distribution of 20 nm (green) microbeads to outflow tissues in the trabecular meshwork and angular aqueous plexus. Therefore, confocal analyses demonstrate comparable distribution in retinal and anterior outflow tissues between perfused and live pig eyes from ITV injection.

As an alternative quantitative analysis, 30 µl aqueous fluid was sampled for anterior segment bead distribution and scanned on a fluorescent plate reader, with mathematical correction for bead size. In the perfusion system, we were able to serially sample from the AC port at 2 h and 4 h. When analysed as a percentage of injected microbeads this resulted in a proportional time-dependent increase in 20 nm (green) beads, while 2000 nm (red) beads remained near threshold (figure 2*e*). For technical reasons we were only able to collect the 4 h sample from live pig eyes, so the raw microbead numbers were compared to 4 h samples from perfused eyes. Both samples showed a similar distribution of 20 nm (green) beads that was not significantly different and a large ratio between 20 nm and 2000 nm beads (figure 2*f*). However, the few 2000 nm (red) beads did show a small, but significant difference between perfused and live samples (*p* = 0.043). Finally, as an important control, non-perfused eyes were also assessed, in which the apparatus was assembled with cannulations, but without activation of the perfusion pump. No signal from either bead size was detectable in these non-perfused control samples, demonstrating that the distribution we observed was dependent on active fluid dynamics in these eyes rather than passive diffusion or buoyancy (figure 2*f*). Together, these experiments demonstrate a size-dependent anterior particle distribution that is conserved between live and perfused pig eyes.

### 3.3. Intraocular particle movements can be assessed pre-clinically in the perfused human eye

One of the most exciting uses for an organotypic perfusion approach is the potential to assess pre-clinical particle and drug disposition in human eyes, and to draw comparisons to surrogate animal models. Therefore, we set up experiments to validate this approach for anterior movements using fresh donated human globes. To maximize the use of available donor tissues, we included eyes that maintained an expanded facility range between 0.4 and 0.18 µl min^−1^/mmHg^−1^ and chose a 4 h time-point for proof-of-principle studies.

**Figure 3.**
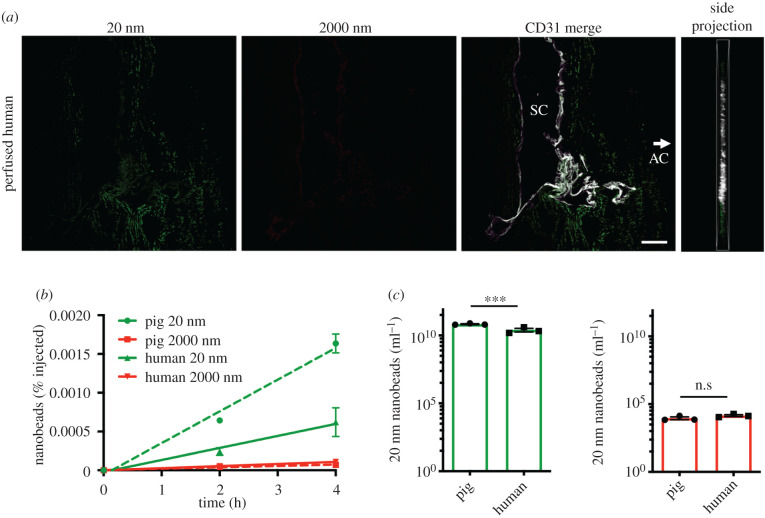
Anterior microbead distribution is conserved in perfused human eyes. (*a*) Representative confocal projections of sectioned human outflow tissues following 4 h perfusion *ex vivo* showed deposition of 20 nm beads (green), but no 2000 nm beads (red) in trabecular meshwork and SC as visualized by CD31 labelling (white, Merge), similar to the pig studies (*n* = 3, arrow indicates the location of the AC for orientation). A side projection indicates beads remained in the tissue plane (Side, *n* = 3). (*b*) Similar to porcine samples, corresponding serial aqueous samples from perfused *ex vivo* human eyes exhibited a time-dependent increase in the percentage of 20 nm microbeads, but 2000 nm microbeads remained near baseline. For comparison, pig perfusion data are also plotted on the graph in dashed lines (*n* = 3, linear regression shown, note; error bars smaller than the data point do not plot). (*c*) Comparisons at 4 h between analysed human and porcine aqueous samples show a similar ratio of raw numbers of microbead sizes, with dramatically higher concentrations of 20 nm beads than 2000 nm. The human perfusions resulted in small, but significantly lower concentrations of 20 nm beads than in the pig (*n* = 3). (Scale bars indicate 50 µm, data are mean ± s.e.m., ***p < 0.001).

Human eyes were injected intravitreally with the same microbead combination as porcine eyes and assessed with parallel methods. Similar to live and perfused pig samples, anterior outflow tissue projections showed 20 nm beads (green) in the trabecular meshwork and surrounding SC, but no 2000 nm beads (red) (figure 3*a*). Serial collection of 30 µl aqueous fluid provided an indirect quantitative comparison of anterior microbead flow in pig and human eyes. Using the 2 h and 4 h time-points from both species for calculation, an anterior dispersion rate of 1.62 × 10^10^ beads ml^−1^ h^−1^ was calculated for 20 nm (green) beads for the perfused pig eye, compared with 6.15 × 10^9^ beads ml^−1^ h^−1^ for human. By contrast, 2000 nm (red) beads migrated at only 2.45 × 10^3^ beads ml^−1^ h^−1^ in the pig eye and 3.59 × 10^3^ beads ml^−1^ h^−1^ in the human eye (figure 3*b*). However, raw microbead numbers show a similar ratio of 20 nm to 2000 nm microbeads between species (figure 3*c*). Therefore, these data suggest that although the trends are similar, there is a significant (2.63-fold) increase in 20 nm anterior particle migration in pig versus human eyes, providing quantitative scaling for particle distribution between species.

### 3.4. Validation for adverse movements of GNE-947 particles in pre-clinical development

**Figure 4.**
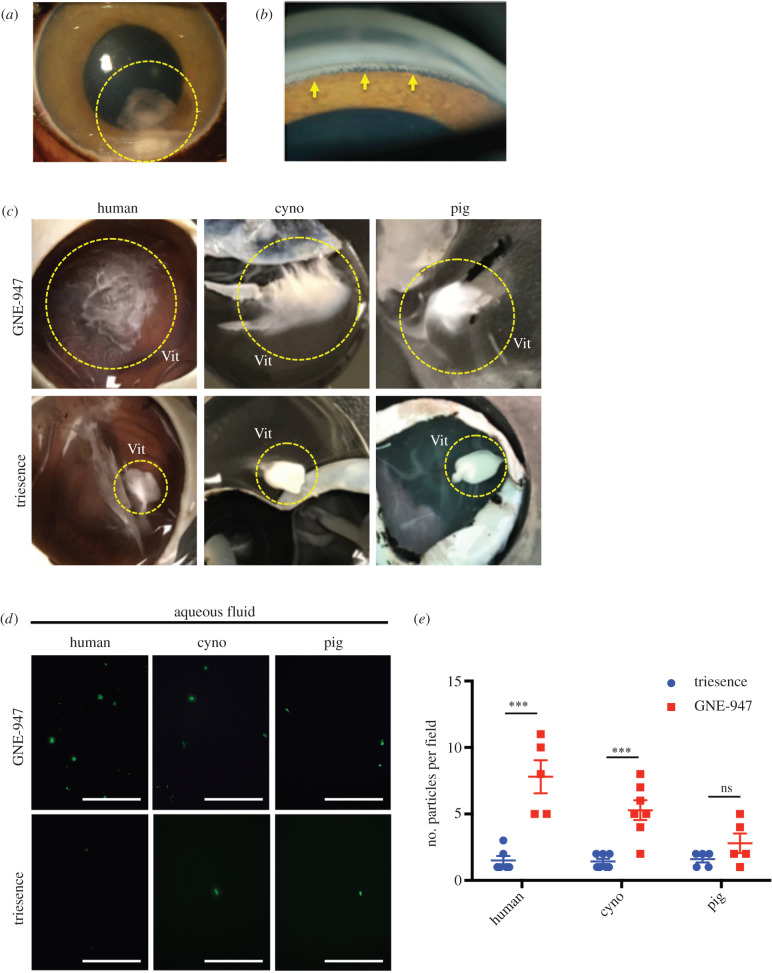
Adverse anterior drug particle movements are recapitulated in perfused NHP and human eyes. (*a*) Representative slit lamp biomicroscopy image of GNE-947 formulation particles in the AC (outlined by yellow dotted line) of NHP eyes 4 days after ITV administration. (*b*) Representative gonioscopic image of GNE-947 particles settled into the filtration angle (yellow arrows). (*c*) After 4 h of perfusion, the GNE-947 formulation deposits (yellow dotted circles) were evaluated in the vitreous chamber (Vit). Deposits appeared diffuse in human and NHP (cyno) eye vitreous, but less so in pig eye vitreous, compared to Triesence, which remained a compact deposit in the vitreous for all species (*n* ≥ 5). (*d*) Representative images of aqueous samples after 4 h of perfusion, spotted on a slide under 488 nm excitation, which display more GNE-947 particles than Triesence (scale bars indicate 50 µm). (*e*) Corresponding particle counts reveal significantly more GNE-947 particles in aqueous than Triesence in human eyes (*n* ≥ 5) and NHP eyes (*n* ≥ 5), but not pig (*n* ≥ 5). (***p < 0.001, ns; not significant). Vit; vitreous body.

The small molecule compound, GNE-947, is a potent PI3 k/mTOR inhibitor developed with an ITV sustained-release suspension formulation for the treatment of intraocular neovascularization. During pre-clinical development, this formulation was tested in an ocular tolerability study in cynomolgus monkeys, where unexpected rapid movement of particulate matter to the AC was observed (figure 4*a*), with particles settling into the angle (figure 4*b*). IOP subsequently increased to above 30 mmHg, presumably owing to particles blocking the outflow angle, elevating the risk of glaucoma. This *in vivo* result presented an opportunity for us to validate our system by recapitulating this experiment *ex vivo*. Specifically, we wished to compare *ex vivo* results using GNE-947 to those using Triesence (triamcinolone acetonide) as a control. Triesence is a marketed ITV suspension with similar formulation components to GNE-947 that has not been observed to migrate anteriorly [11].

Each formulation was tested in our perfusion system by ITV injection in human, cynomolgus and pig eyes using a four-hour time-point, as previously established for microbeads. Following perfusion, eyes were hemisected and the posterior segment and intact vitreous were analysed under low magnification to inspect the injected bolus. The GNE-947 pellet was noticeably more diffuse in all cases than Triesence, which retained a compact appearance (figure 4*c*). In order to quantify the particles migrating to the AC, aqueous humour was sampled from the same eyes and assessed by confocal microscopy. Particles in each formulation fluoresced upon excitation at 488 nm, providing a convenient method for distinguishing drug particles (electronic supplementary material, figure S5). Quantification of confocal images (figure 4*d*) revealed significantly more particles of GNE-947 than Triescence in simulated aqueous humour samples from human and cynomolgus eyes (figure 4*e*). In comparison, there was a similar trend in pig eyes, but the difference between formulations did not reach significance (figure 4*e*). Therefore, the *ex vivo* human and cynomolgus results were consistent with the *in vivo* cynomolgus observations.

### 3.5. Retinal particle distribution shows comparable distribution in live versus perfused pig eyes and can also be assessed pre-clinically in perfused human eyes

**Figure 5.**
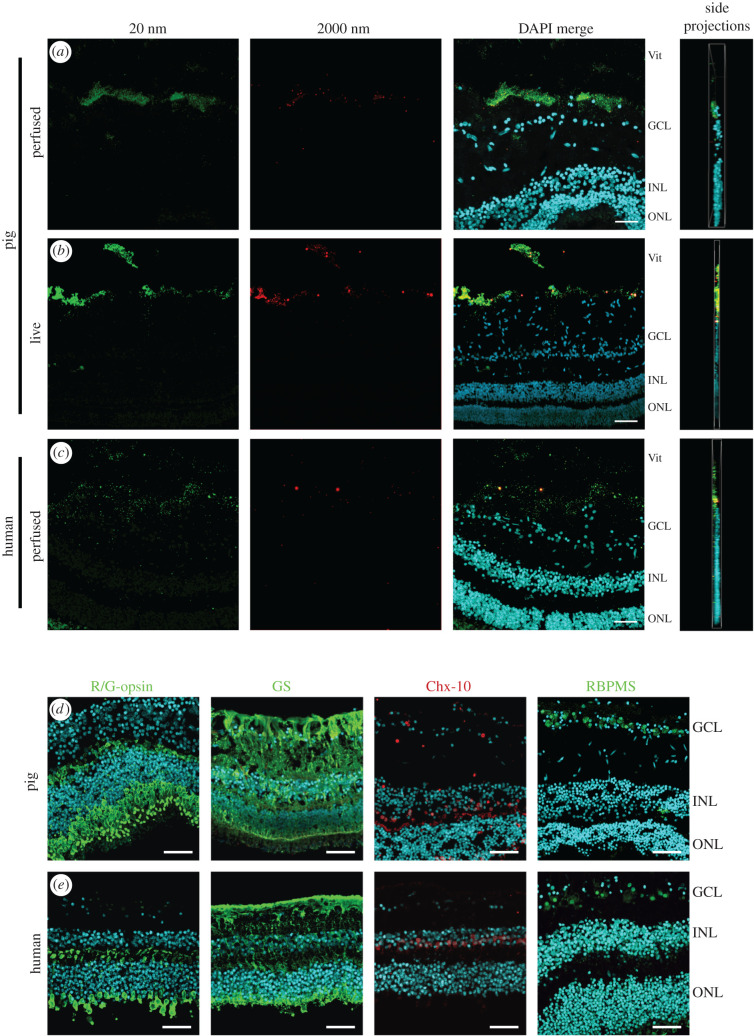
Retinal microbead distribution are preserved in perfused human and porcine eyes. Confocal projections of retinal sections were assessed from regions underlying the microbead pellet from perfused and live pig eyes, and *ex vivo* human eyes after 4 h perfusion. (*a*) Perfused pig retina images demonstrated the distribution of 20 nm beads (green) and fewer 2000 nm beads (red), with microbeads of both sizes generally excluded from penetrating into the retinal layers (indicated by text along the right-hand side of the merged image). A side projection was also assessed to ensure beads remained in the tissue plane (*n* = 3). Comparable retinal microbead distribution patterns were observed at 4 h following injections in (*b*) live pig eyes (*n* = 3) and (*c*) perfused human eyes (*n* = 3), with very few of either size penetrating into the retinal layers. (Scale bars indicate 50 µm). (*d*) Representative confocal images of central retina sections from *ex vivo* pig eyes following 4 h perfusion, stained with antibodies to the markers R/G opsin, GS, Chx-10 and RBPMS (*n* = 3, Scale bars indicate 50 μm, note the sections are oriented the same way, with the retinal layers indicated along with the right-hand panel). (*e*) Representative confocal images of central retina sections from *ex vivo* human eyes following 4 h perfusion, stained for the same markers as in (*d*) (*n* = 3, Scale bars indicate 50 µm, retinal layers are indicated along with the right-hand panel). GCL; ganglion cell layer, INL; inner nuclear layer, ONL; outer nuclear layer, Vit; vitreous.

Based on our promising vitreous and anterior segment results, we wondered whether this perfusion approach could also be used to study the distribution of ITV formulations to the retina. To assess inner retinal particle distribution, sections from microbead-injected human and pig eyes were carefully prepared and analysed by confocal microscopy. Both *in vivo* and *ex vivo* perfused pig sections showed accumulation of 20 nm (green) and 2000 nm (red) microbeads adjacent to the inner retina at regions underlying the ITV injection site (figure 5*a,b*). Previous literature on pore sizes of the inner limiting membrane (ILM) suggests that it is permeable to particles of 10–100 nm [65,67,68]. Interestingly, both sizes of microbeads were generally excluded from penetrating the retinal layers in *in vivo* and *ex vivo* pig eyes after 4 h (figure 5*a,b*). Perfused human eyes were also assessed at the retinal region adjacent to the injection site and displayed similar results, with both 20 nm and 2000 nm microbeads not penetrating the retinal layers (figure 5*c*). To confirm that retinal cytoarchitecture remained intact during perfusion, pig and human retinas were also probed with a panel of antibodies to cellular markers for cone photoreceptors (red-green opsin; R/G-opsin), Müller glia (GS), bipolar cells (Chx-10) and ganglion cells (RBPMS) (figure 5*d,e*). These markers indicate broad preservation of retinal cytoarchitecture following perfusion. Together these data demonstrate the potential to assess retinal particle distribution in this model. Furthermore, the exclusion of microbeads from penetrating into the retina suggests that key tissue barriers, such as the ILM, remained intact during perfusion.

### 3.6. Considering the vitreous humour as a poroelastic medium is consistent with significant bidirectional fluid transport away from the intravitreal injection site

The observation of ITV particles reaching both the retina and trabecular meshwork indicates a bidirectional flow of fluid and convective particle transport away from the injection site in the vitreous humour. This in turn suggests that the vitreous is locally pressurized to drive fluid flow (and particle transport) away from the injection site. We wondered whether these observations are consistent with other similar phenomena. Indeed, such a mechanism has been considered in other contexts, notably during fluid infusion into the brain [69]. Briefly, an initial fluid injection locally pressurizes the tissue into which the injection occurs in a manner that depends on the injected volume and the bulk modulus of elasticity of the tissue. This is a classic problem in porous media theory [70,71], which can be described by the so-called Biot model of consolidation. The consolidation (local volume change of the porous material) is associated with a local change in fluid pressure; pressure and volume relax back to their baseline value as fluid is transported within the porous material after a local perturbation, e.g. a fluid injection.

Basser has considered this problem for the brain in some detail and gives mathematical expressions for transient pressure, flow and consolidation profiles after a step change in fluid infusion [69]. It is instructive to consider the time constant associated with fluid transport following a step increase in pressure at an injection site. This time constant, denoted by τ, represents a characteristic time for advection to relax back to its equilibrium state after an injection and is given by

$$\tau =\frac{\;f\;{\left(R_{o}-a\right)}^{3}}{a\kappa P_{o}},$$

where *f* is the fluid volume fraction in the tissue (taken as approximately 1 for the highly porous vitreous humour); *R*_0_ is the radius of the porous material in which advection is occurring, i.e. the vitreous humour (taken as 1.2 cm for a porcine or human eye); *a* is the radius of the injected fluid volume (taken as 0.23 cm for a 50 microliter injection), κ is the hydraulic permeability of the vitreous humour and *P*_0_ is the initial pressure at the injection site owing to the injection. κ has been measured for bovine vitreous humour as $8.4\times 10^{-8}$ cm^4^/(dynes) [32]. The injection pressure *P*_0_ was not measured in our experiments, but can be roughly estimated by using the value of the ocular rigidity [72], which states that IOP increases by approximately 1 mmHg for each microliter volume change of the eye, suggesting a lower bound value of *P*_0_ of 50 mmHg, or $6.7\times 10^{4}$ dynes cm^−^^2^. Substituting these numerical values into the above equation, we obtain a value for τ of 705 s. In other words, the trans-vitreal flow arising from the initial injection is estimated to persist for several tens of minutes. We comment that the above analysis is only approximate: the derivations of Basser were for an infinite porous material, yet the eye has finite size and thus an intraocular injection will lead to scleral expansion and a pressure elevation throughout the eye. The unsteady advective process we have described above, with time constant τ, is in addition to the above-mentioned global pressure rise owing to scleral expansion. Clearly this situation is complex and a complete analysis would require the theory of Basser to be extended to the case of a finite domain bounded by an elastic shell, which is outside the scope of the present work.

It is intriguing to next ask what magnitude of fluid velocity is induced within the vitreous humour by this injection, *V*_0_. Using Darcy's law, we can estimate that a local pressure elevation *P*_0_ within a region of characteristic size *R*_0_ will produce velocities of order $V_{0}∼(\kappa P_{0}/R_{0})=4.7\times 10^{-3}$ cm s^−1^. The product of τ and *V*_0_ gives an approximate length scale, *L*_0_, over which fluid can be transported before the pressure transient dissipates. Using the above values, we obtain the surprisingly large value of *L*_0_ = 3.3 cm. This result supports the concept that intravitreally injected fluid can move bidirectionally to reach both the retina and anterior segment.

## 4. Discussion

The major finding of this work is that a simple *ex vivo* perfused eye model can replicate important aspects of particle transport after ITV injection, which we foresee as having great use in pre-clinical evaluation of drugs to treat a variety of ocular conditions. This work was motivated by the many challenges in predicting intraocular drug disposition, which stem from the anatomy of the eye itself, as a compartmentalized organ consisting of discrete, but interconnected, chambers. The anterior and posterior chambers of the anterior segment contain aqueous humour fluid, while the much larger posterior segment is filled with a combination of fluid and vitreous humour [73,74]. The enclosed nature of the eye makes local delivery an attractive strategy, with a wide variety of sustained-release formulations and strategies in various stages of pre-clinical development [75–80]. Yet, dynamic fluid flow between the ocular compartments, anatomical differences between species and age-related vitreous liquefaction combine to form a complex system in which particle movements can be difficult to measure and predict *in vivo*. Similarly, the comparative nature and role of intraocular barriers in multiple species require further characterization. Finally, clinical sampling of intraocular fluid or tissues are often not possible, resulting in an inability to directly assess drug behaviours in the human eye. Therefore, literature on particle distribution in human eyes remains surprisingly sparse, and the development of laboratory models to predict and evaluate these parameters pre-clinically is of critical importance.

The primary goal of the organotypic perfusion approach was to re-establish whole-globe IOP, temperature gradient and fluid dynamics, in order to assess the anterior movement of particles in ITV-injected human or animal eyes. This strategy exhibits several advantages by recapitulating anterior outflow dynamics [40–43], while also retaining anatomical barriers between intraocular compartments and allowing direct comparison between ITV-injected material in an animal model and human eyes. Stable pressure/outflow facility in this context was monitored to indicate stable fluid convection and ocular integrity. However, it is important to note that the influence of EVP and retinal vascular flow and leakage, as well as daily fluctuations in IOP, is not represented in this model at present. Similarly, the potential influence of subretinal fluid pumping by the retinal pigmented epithelium has not yet been assessed [81]. EVP makes important contributions to IOP *in vivo* and is a key term of the modified Goldmann equation used to calculate outflow [82,83]. However, a primary advantage of the present approach is that it simplifies these effects by artificially generating a constant flow to result in physiological IOPs and avoids complex vascular considerations [43,52]. In the present study, the absence of the EVP component, or of small fluctuations in IOP, did not result in significant differences in the evaluated parameters between live and perfused eyes. However, the influence of such fluctuations could be modelled in future refinements. In comparison, perfusion systems cannulating the posterior ciliary artery have been previously reported in bovine and porcine eyes in order to predict the disposition of ITV formulations [47–50]. Yet, these models do not generate defined intraocular flow, nor are they possible in human eyes owing to inconsistent and irreversible coagulation during the post enucleation period.

Alternatively, in order to model human eyes, several three-dimensional physiologically based computational models have been proposed to predict the disposition of ITV formulations, which capture aspects of ocular anatomy and vitreous outflow [34,36,84–86]. However, this literature only partially captures ocular anatomy and has had to rely on limited datasets for calibration with little opportunity for follow-up validation or refinement [36,85,87]. Also, many such models are calibrated or anatomically based on the rabbit eye; a common experimental system for testing ophthalmic drugs, but with a highly viscous vitreous and different aqueous outflow and anatomy compared to human and NHP eyes [28–30]. We propose that this perfusion approach can be used to provide further detail on the differences between ITV distribution between species. An initial comparison of relative AH_syn_ concentrations of the test articles used between species in the present study suggests modest differences in scaling, particularly between human and pig perfusions (electronic supplementary material, table S1). However, considerable additional particle movement data will be needed to facilitate the construction of more comprehensive anatomic and scaling models for particle transport, validated directly against human eye data.

Here, we have tested the hypothesis that re-establishing homeostatic aqueous dynamics will drive directional ITV particle movements in a physiological manner. To validate this approach, anterior particle movements of ITV-injected particles showed generally consistent behaviours between large and small particles in live and perfused pig, and perfused human eyes. However, the significant difference in anterior bead distribution rate in pig versus human eyes suggests the impact of additional physical and anatomical variables that require further assessment. Interestingly, gravity and eye orientation had no significant impact on this behaviour, as the live pig and monkey experiments necessarily involved dynamic and horizontal orientations, while the perfusion system was kept in a vertical orientation for simplicity and to be consistent with previous methodologies for measurement of outflow facility. The active and flow-driven nature of the results are underscored by the negative controls performed without perfusion that showed little anterior particle movement, thus excluding passive diffusion as a major contributor to this behaviour [79]. Additionally, the bidirectional distribution of particles to the inner retina suggests that a posterior flow must also be present. Interestingly, a simple poroelastic model of fluid transport in the injected eye supports the feasibility that intravitreally injected fluid can easily reach both the retina and anterior segment. Of course, the transport of particles and macromolecules will be hindered as compared to fluid transport, so that their transport distances will be smaller depending on parameters such as macromolecular size. However, the basic message remains: a simple order-of-magnitude analysis indicates that injected materials can disperse widely within the eye over the time scale of a few tens of minutes. This same process would be expected to occur *in vivo*, and we suggest that these analyses may provide the basis for a useful mechanistic understanding of transport within the eye following an ITV injection.

Our validation study with GNE-947 and Triesence suggests that formulations in the pig eye may exhibit distinct behaviours from the human and cynomolgus, possibly owing to anatomical factors such as vitreous viscosity and intraocular barriers. There were also significant differences in particle distribution and movement between GNE-947 and Triesence, both *in vivo* and in the *ex vivo* model, despite relatively subtle differences in formulation characteristics, with no differences in charge between major components. These results, together with our microbead results and previous analyses [64], are consistent with an aggregate particle size as an important parameter governing vitreous distribution. Also of note were the similar retinal particle distributions we observed between species, and the surprisingly well-preserved retinal cytoarchitecture suggests the further potential to employ this strategy for optimizations of retinal drug penetration from ITV administration. Interestingly, Schargus *et al.* [88] recently reported on parameters influencing variable particle burden and aggregates in clinical anti-vascular endothelial growth factor drug formulations that could similarly be compared for their influence on distribution in our model. Thus, the successful design of particle-based delivery systems for intraocular applications could benefit from using this organotypic approach to pursue further assessment of attributes governing particle distribution in pre-clinical human eyes or model species.

## Data accessibility

The main data supporting the findings of this study are available within the article and its electronic supplementary material. The raw data generated in this study are available from the corresponding author upon reasonable request.
